# How AI Systems Can Be Blameworthy

**DOI:** 10.1007/s11406-024-00779-5

**Published:** 2024-10-01

**Authors:** Hannah Altehenger, Leonhard Menges, Peter Schulte

**Affiliations:** 1 Department of Philosophy, University of Konstanz, Universitätsstraße 10, 78464 Konstanz, Germany; 2 Department of Philosophy (Faculty of Social Sciences), University of Salzburg, Franziskanergasse 1, 5020 Salzburg, Austria; 3 Department of Historical, Philosophical and Religious Studies, Umeå University, Universitetstorget 4, 901 87 Umeå, Sweden

**Keywords:** Artificial Intelligence, Robots, Blameworthiness, Responsibility, Quality of Will, Attributability, Desire

## Abstract

AI systems, like self-driving cars, healthcare robots, or Autonomous Weapon Systems, already play an increasingly important role in our lives and will do so to an even greater extent in the near future. This raises a fundamental philosophical question: who is morally responsible when such systems cause unjustified harm? In the paper, we argue for the admittedly surprising claim that some of these systems can themselves be morally responsible for their conduct in an important and everyday sense of the term—the attributability sense. More specifically, relying on work by Nomy Arpaly and Timothy Schroeder (*In Praise of Desire*, OUP 2014), we propose that the behavior of these systems can manifest their ‘quality of will’ and thus be regarded as something they can be blameworthy for. We develop this position in detail, justify some of its crucial presuppositions, and defend it against potential objections.

## Introduction

AI systems play an increasingly important role in our lives. They have taken over many jobs that we used to do ourselves. They already play significant roles in various important domains, such as, for example, transportation, warfare, and medical care, and they will increasingly do so in the years to come.

These systems, which either already exist or will, with high probability, exist in the near future (henceforth “realistic AI systems”) are not the human-like beings of mainstream science fiction, like Data or C-3PO. Still, they are clearly more autonomous than traditional machines (at least in some sense of the word ‘autonomous’). They move about, process information about their environment, make decisions (at least in some sense of the word ‘decisions’), and learn to do things they were not directly programmed to do. Thus, it seems that with them, a new kind of agent has arrived on the scene.

This is not only important from a sociological or legal point of view; it also raises some fundamental philosophical questions. Among other things, the following question arises: can realistic AI systems be morally responsible for their conduct, or do they belong to the class of agents who are exempted from moral responsibility such as, for instance, non-human animals or toddlers?

In the following, we take on this intriguing issue. Our main claim is that some realistic AI systems can indeed be blameworthy for their conduct in a philosophically well-established and non-metaphorical sense of the term—the attributability sense. Drawing on recent developments in responsibility scholarship, we will argue more specifically that some realistic AI systems can express a problematic quality of will through their conduct, i.e., a lack of good will or even ill will, and that this is sufficient for an interesting and important form of blameworthiness.

We are not the first to argue that some realistic AI systems can be blameworthy or responsible for their conduct: to give a few examples, Laukyte (2014, 2017) and Christian List (2021) have arrived at the same conclusion by emphasizing an analogy between artificial agents and group agents. Floridi (2016), focusing on actions stemming from a network of agents which may include both humans and realistic AI systems, has argued that responsibility (understood as ‘faultless responsibility’ or strict liability) can be attributed to *all* agents in the network. Moreover, Tigard (2021a) has recently made a case for the claim that realistic AI systems can be blameworthy in the answerability sense. By focusing on *attributability*, we seek to make another contribution to this increasingly rich landscape of accounts of AI-blameworthiness and responsibility.

We will proceed as follows. In Section 2, we present the basic idea of the quality of will approach to blameworthiness. In this context, we also clarify how the position that we shall defend in this paper relates to the so-called responsibility gap debate, which has recently received much attention in AI ethics. In Section 3, we make an initial case for the claim that the behavior of some realistic AI systems can be explained by a lack of good will and, therefore, be regarded as something these systems are blameworthy for. Section 4 spells out the details. Specifically, our proposal will be relying on the ‘desire-based’ quality of will account of blameworthiness that has been defended by Nomy Arpaly and Timothy Schroeder (2014).^1^ In Section 5, we will defend the resulting view against objections. Finally, Section 6 concludes the discussion.

## Preliminaries

### The Kind of Blameworthiness We Are Concerned With

In a first step, we need to clarify how we understand the notion of blameworthiness. In this paper we will focus exclusively on what is sometimes called blameworthiness in the attributability sense. Blameworthiness in this sense is fundamentally concerned with whether it is fitting or warranted (we will use these words interchangeably) to attribute negative agential properties to someone on the basis of their conduct (see Watson, 1996; Arpaly and Schroeder, 2014, Chap. 7; Shoemaker, 2015, Chap. 1).

Some authors argue that attributability is the *only* kind of blameworthiness and responsibility there is (e.g. Smith, 2012; Talbert, 2022). They hold that if an action is, in the relevant sense, attributable to an agent then that agent is blameworthy (or responsible) for the action in every interesting sense of “being blameworthy” (or “being responsible”). Other authors, such as Watson (1996), Arpaly and Schroeder (2014, section 7.1), and Shoemaker (2015), by contrast, suggest or argue that agents can be blameworthy (and responsible) in the attributability sense without being blameworthy (and responsible) in other senses.^2^ In principle, our main claim that realistic AI systems can be blameworthy in the attributability sense is compatible with both positions. In light of considerations that we will discuss in Section 5, we shall assume, however, that attributability is just one kind of blameworthiness (and responsibility).^3^

To illustrate this kind of blameworthiness, imagine that Abegail steps on your foot simply because she was pushed, and that Babila steps on your foot simply because she wants to hurt you. In all other relevant respects, the situations are equal. Your physical pain is the same, but you would probably respond quite differently to Babila than to Abegail when you learned why they stepped on your foot. You may think of Babila as cruel and disapprove of her conduct. But you would not respond in these ways to Abegail. This difference in responses looks fitting: Babila’s stepping on your foot expresses something morally relevant about her such that morally evaluating her based on her conduct seems correct and it seems warranted to disapprove of her cruelty. By contrast, Abegail’s stepping on your foot does not express anything morally relevant about her. Therefore, a moral evaluation of her based on her stepping on your foot seems unfitting.

It is standardly assumed that being responsible for one’s behavior in the attributability sense presupposes having a *quality of will*.^4^ Thus, Abegail and Babila are only responsible for their conduct in the attributability sense if they have the capacities or other agential features that are necessary for having a quality of will. More specifically, on this approach, actions can express the quality of will of the agent. If an action expresses *good will*, then a *positive moral evaluation* and *approval* of the agent and her conduct is warranted and the agent is *praiseworthy* in the attributability sense. If, by contrast, an action expresses *a lack of good will* or even *ill will*, as Babila’s action arguably does, then a *negative moral evaluation* and *disapproval* of the agent and her conduct is warranted, and the agent is *blameworthy* in the attributability sense.

Our main thesis for which we shall argue in the remainder of the paper is that some realistic AI systems can be blameworthy in the attributability sense, since their conduct can express a lack of good will (and, in some cases, even the presence of ill will). But before moving on to this, let us briefly comment on two issues: why attributability is an important form of responsibility and blameworthiness, and how the position which we shall defend in the following fits in with the debate about the so-called *responsibility gap*.

### The Importance of (AI) Attributability

At least since Gary Watson’s seminal “Two Faces of Responsibility” (1996), attributability has been recognized and discussed as an independent sense of responsibility. It has figured in many influential treatments and is nowadays widely accepted as an important form of responsibility.^5^ Moreover, even disregarding the fact that attributability is of great interest to current responsibility scholarship, this form of responsibility also plays an important role in our everyday lives. This is because the kind of blame that is warranted if an agent is blameworthy in the attributability sense can fulfill several valuable functions.

To see this, let us again take up the case of Babila, who steps on your foot simply because she wants to hurt you and whose action thus expresses a lack of good will. Babila is blameworthy in the attributability sense, and it is therefore fitting to assess her as cruel and to disapprove of her behavior. We can think of this complex response—consisting of a negative moral evaluation of the agent and a disapproving attitude—as a form of blame that we will call “attributability blame”. 6 Note that this response does not involve reactive emotions, such as anger, resentment, or indignation that are often taken to be essential for other kinds of blame (for overviews see Tognazzini and Coates 2018; Menges, 2023).^7^ Still, in every day life, it would be fine to call this response “blame”.^8^

Attributability blame can fulfill at least three valuable functions or, equivalently, can play at least three important roles (for a similar line of reasoning, see also Altehenger and Menges 2024).^9^ First, it can serve the function of protesting against an agent’s conduct (see Talbert, 2012, Smith, 2013). When we protest agents’ conduct, we, thereby, make it clear to ourselves and to others that we do not accept what they did. When, for instance, we assess Babila’s behavior as cruel and disapprove of her cruelty, this plays the important role of making it clear to ourselves and, when expressed, to others that we do not accept her conduct.

Second, attributability blame can signal our commitment to certain norms and values (see Shoemaker and Vargas 2021). When we evaluate Babila’s behavior as cruel and disapprove of her cruelty, we, thereby, signal to ourselves and, when we express it, to others that we have internalized certain norms that she has breached or that we have certain values that she disrespects. Moreover, we, thereby, signal our commitment to enforce these norms or protect these values by, at least, criticizing those who violate or disrespect them.

Third, attributability blame can initiate conversations about the moral status of Babila’s conduct (see McGeer, 2013). When we respond to Babila’s stepping on your foot by assessing her as cruel and disapproving of her behavior, then this response can be the starting point of an *intra*personal conversation: we may ask ourselves what reasons she may have to act this way and what reasons we have to be against it. When we express our blame response to Babila, and start a conversation *with her*, then this may help her see what reasons she has and how her moral community perceives her. But even if it should be impossible to have such a moral conversation with Babila, blaming her can still fulfill another important function: the expressed blame response can initiate a conversation *with third parties* about her conduct and the status of her cruelty (see also Altehenger and Menges 2024).All these kinds of conversations can help us to develop and foster our capacities to respond to moral reasons.

We have recently argued that these valuable functions can still be fulfilled when the receiver of such responses is a realistic AI system (see Altehenger and Menges 2024). But we  have not yet shown that realistic AI systems are *blameworthy* in the attributability sense. In the following, we want to close this gap.

Against this approach, one might raise the following objection: 10 engaging in protest, signaling, or normative conversations can be justified on purely instrumental grounds, regardless of the target’s blameworthiness. Thus, if the functions of blaming realistic AI systems in the attributability sense are (merely) to protest, signal commitment, and initiate moral conversations, then it seems irrelevant whether these systems are, in fact, blameworthy.

Let us briefly present two replies. First, answering the question of whether realistic AI systems are blameworthy in the attributability sense is important because it may advance certain debates in AI ethics (as we shall show in more detail in the following sub-section). Second, answering this question is also important when we ask ourselves if blaming realistic AI systems is appropriate. To see this, note that blame can be appropriate in different senses. According to one sense, blaming a target is appropriate if it is *overall justified*. Then, the reasons against blaming the target are not stronger than the reasons for it. Admittedly, blaming a target can be appropriate in this sense even if the target is not blameworthy. However, we may also want to know if blaming a target is appropriate in the sense that *nothing speaks against it* (henceforth called *impeccable*). Blaming a target that is not blameworthy is, surely, not impeccable. It would be unfitting, just as it would be unfitting to desire something undesirable or to be amused by a joke that is not funny. Thus, when we want to know whether blaming a target is impeccable, then we need to know if the target is, in fact, blameworthy in some relevant sense of the term. Moreover, note that ethicists often focus on features that count against or for certain responses even if these features do not directly determine overall justification. And insofar it speaks against blaming a target that the target is not blameworthy, this paper is concerned with something ethically important.

### Attributability and the Responsibility Gap Debate

Next, let us clarify how the position which we shall defend in the following fits in with the responsibility gap debate. The responsibility gap debate starts from the assumption that some realistic AI systems are so ‘autonomous’ that neither the designers nor the users have the kind of control over or knowledge about their behavior that could ground the users’ or designers’ responsibility for what these systems do. To this, the further assumption is added that the systems themselves cannot be responsible for their behavior. Hence, a responsibility gap arises: nobody (or nothing) seems to be responsible for what these systems do even if they cause severe unjustified harm (for different views on the matter see e.g., Matthias, 2004; Sparrow, 2007; Himmelreich, 2019; Nyholm, 2020; Köhler 2020; Tigard 2021b; Danaher, 2022; Kiener, 2022; Königs 2022; for an overview see Müller, 2020).

Now, different authors seem to have different things in mind when they use the words ‘responsibility’ or ‘blameworthiness’ in the responsibility gap debate. Solum (1992, 1244-48) and Sparrow (2007, 72), for example, are mainly concerned with the question of whether AI systems can deserve punishment (see also Danaher, 2016). Kiener (2022, Section 3), by contrast, is concerned with responsibility as answerability, which he understands as the obligation to explain and justify conduct (for a slightly different view on answerability see Tigard, 2021a).

As the preceding made clear (Section 2.1 and 2.2), blameworthiness in the sense we are interested in differs both from deserved punishment and an obligation to justify one’s conduct. It makes full sense to assess an agent as cruel and disapprove of her conduct, and to simultaneously maintain that she does not deserve punishment (perhaps because one is skeptical about deserved punishment in general) or that she is not obliged to justify her conduct (perhaps because she should focus on something more important). That is, an agent’s being responsible in the attributability sense neither implies responsibility as ‘punishability’ nor responsibility as answerability in Kiener’s sense.

Hence, the question of how the proposal we shall put forward in this paper fits in with the responsibility gap debate can ultimately only be answered in a conditional way: if one holds the view that the responsibility gap debate is about whom to punish or about who is obligated to provide an explanation, then the reasoning we offer in the following cannot contribute to closing responsibility gaps. By contrast, if one holds the view that the responsibility gap debate is about who is morally responsible *in some important sense of the term*, then the account we shall offer in the following might provide additional resources for closing responsibility gaps, since we defend the claim that there is an important sense in which some realistic AI systems can themselves be morally responsible when they cause unjustified harm (the attributability sense).

To sum up, this paper is concerned with the question of whether realistic AI systems can be blameworthy in an important and non-metaphorical sense—the attributability sense. In order to answer this question, we need to examine whether realistic AI systems can have and express a certain quality of will. In the following sections, we will argue that they can.

## An Initial Case for the Blameworthiness of Realistic AI Systems

Here are two cases which suggest that the behavior of some realistic AI systems can express a lack of good will.

**Human Taxi Driver**: Travis is an SUV taxi driver whose main goal when driving his taxi is to maximize profit. Travis has learned that the best way to do this is to drive as quickly as possible to the customers and to drive them as quickly as possible to their destinations. Travis has also learned that, in most situations, following traffic rules is helpful for achieving his main goal. But when nobody is watching, breaking the rules can be more efficient. Right now, Travis is driving through a long and narrow lane when he realizes that a man is lying in the street. The customers are deep in conversation and aren’t paying attention to anything outside. It is dark outside and nobody is watching. Travis realizes that running the man over is the most efficient way to achieve his main goal: driving around the man is not possible. Turning around, or stopping the car, would mean a significant delay. And nobody will learn about it if he runs the man over. Consequently, Travis goes on to do it.

Plausibly, Travis has the moral obligation to stop and help the human in the street. However, Travis is indifferent to moral considerations. He only cares about maximizing profit and, accordingly, reaching his destinations as quickly as possible.^11^ Travis’ conduct is a manifestation of ruthlessness and selfishness such that attributing these properties to him on the basis of his conduct and disapproving of his action is warranted. In this sense, Travis is blameworthy for this conduct. Now consider

**Machine Taxi Driver**: Tesber is a self-driving SUV taxi whose main goal when on the road is to maximize profit. Tesber has learned that, in most situations, following traffic rules is helpful for achieving its goal. But when nobody is watching, breaking the rules in order to be faster is more efficient. Right now, Tesber is driving through a long and narrow lane. The customers are deep in conversation and aren’t paying attention to anything outside. It is dark outside and nobody is watching. These circumstances are registered by Tesber, who regularly monitors the passengers as well as its immediate surroundings. Right now, Tesber detects a man who is lying in the street. From the available information, Tesber infers that running the man over is the most efficient way to achieve its main goal: driving around the man is not possible. Turning around, or stopping, would mean a significant delay. And nobody will learn about it if Tesber runs the man over. Consequently, Tesber goes on to do it.

The two cases, thus described, look very similar to each other. It seems as if Tesber, just as Travis, is indifferent to moral considerations. And since the fact that Travis does not care about moral considerations, and acts accordingly, appears to be enough to establish his blameworthiness, it seems that Tesber must be as blameworthy as Travis.^12^

One may block this implication by arguing that it is not true that Travis’ being indifferent to moral considerations makes him blameworthy. Or one may argue that there is a difference between the two cases which explains that Travis is blameworthy, while Tesber is not. In what follows, we will assume that Travis is blameworthy because, due to the fact that he is indifferent to moral considerations, he displays a lack of good will. Therefore, only the second option remains for those who want to argue that Tesber is not blameworthy.

Some may say that the decisive difference between the two cases is that talking about “not caring about” or “being indifferent to moral considerations”, and, in consequence, “lacking good will” is merely metaphorical in Machine Taxi Driver, but non-metaphorical in Human Taxi Driver (see, e.g., Tigard, 2021b, 602). In the following sections, we will argue that this is not so. More specifically, the reasoning that we will offer in support of this claim encompasses two main steps:

First, relying on previous work by Arpaly and Schroeder (2014), we will translate talk of “not caring about” or “being indifferent to moral considerations” into talk of lacking certain intrinsic desires, namely desires for the right (or good), and contend that the fact that an agent’s behavior can be explained by the fact that it lacks such desires is sufficient for its lacking good will (and thus for its being blameworthy).

Secondly, we will argue for the claim that in cases like Tesber’s, the harmful behavior displayed by a realistic AI system can be explained by the fact that the system lacks (certain) intrinsic desires for the right (or good). Hence, the system can be said to manifest a lack of good will, and thus be blameworthy for its conduct.^13^

## Blameworthiness for Realistic AI Systems

One intuitive way to state the core idea of quality of will accounts on which we implicitly relied in presenting the cases of Human Taxi Driver/Machine Taxi Driver is that an agent’s quality of will is a matter of what she cares about (or what is important to her) or, more pertinent to our example, what she fails to care about (or is indifferent to). But when does *S* care about something, or when does *S* fail to care about something?

### The Desire-based Quality of Will Account of Blameworthiness

According to one version of the quality of will approach—the account developed by Arpaly and Schroeder (2014)—, *S* cares about something just in case *S intrinsically desires* it, and *S* fails to care about something just in case *S lacks an intrinsic desire* for it.

Building on the idea just stated, Arpaly and Schroeder (2014) propose (what may be called) a *desire-based quality of will account of blameworthiness*. At the core of this account is the following claim:

Blameworthiness: if *S*’s behavior *x* is explained (through rationalization) (a) by the fact that *S* has an intrinsic desire for the complete or partial wrong or bad (correctly conceptualized) or (b) by the fact that *S* lacks an intrinsic desire for the complete or partial right or good (correctly conceptualized), then *S* is blameworthy for *x*.^14^

In our view, Blameworthiness amounts to an attractive way of spelling out the quality of will approach to blameworthiness, and we will, therefore, presuppose it for the purposes of this paper. Admittedly, Blameworthiness is controversial, and we cannot fully defend it here. However, we will further elaborate on its key components in the course of this section and defend it against two important objections at a later point in the paper (see Section 5.1 and 5.2).

There are several more-or-less close cousins to the desire-based quality of will account of blameworthiness. These include, among others, the accounts defended by David Shoemaker and Chandra Sripada. These views, too, say that if a bad action expresses an agent’s cares, then the agent is, in a certain sense, blameworthy and responsible for it (Shoemaker, 2015, 55; Sripada, 2016, Section 2.2). However, they differ from the desire-based account in how they understand the notion of caring. Rather than translating talk of caring into talk of (intrinsically) desiring, these accounts identify caring with a disposition to have certain emotions (Shoemaker, 2015, 24 − 25) or with a complex conative state that has, among other things, a close connection to an agent’s normative judgments (Sripada 2016, Section 2.2). Somewhat more distant cousins of the desired-based quality of will account ground agents’ quality of will, and hence their blameworthiness, in their normative judgments (e.g., Scanlon, 2008, Smith 2015, Talbert, 2008 and, 2022). Since we neither want to commit to the claim that realistic AI systems can have emotions nor to the claim that they can make normative judgments, we shall leave these alternative views aside and focus exclusively on the desire-based quality of will account of blameworthiness. It is important to note, however, that if it should turn out that realistic AI systems can make normative judgments or have emotions (and the relevant emotional dispositions), then these alternatives to the desire-based account would also suggest that AI systems can be blameworthy.^15^

The remainder of this section is organized as follows: we will first explore whether AI systems like Tesber can have intrinsic desires at all and we will argue that they can (Section 4.2). Then we will show that Tesber lacks an intrinsic desire for the right (or good) correctly conceptualized (Section 4.3), and argue that the absence of such a desire explains Tesber’s conduct (Section 4.4), thus arriving at the conclusion that Tesber is blameworthy for its conduct.

### Realistic AI Systems Can Have Intrinsic Desires

What are intrinsic desires? In this section, we will look at the four main views of (intrinsic) desires that have been defended in the literature.^16^ We will argue that a close examination of these views suggests that we have, on the whole, good reasons to accept the claim that realistic AI systems can indeed have intrinsic desires. Or, at the very least, it shows that we have good reasons to take this claim seriously.^17^

Before we come to our argument, however, we want to forestall a possible misunderstanding. In arguing for the claim that realistic AI systems can have desires, it may seem that we are arguing for a highly ambitious thesis: namely, the claim that such systems can “have minds”. But this way of putting things, while not wholly incorrect, is at least highly misleading. While our claim that realistic AI systems can have desires, given that desires are mental states, entails that realistic AI systems can have minds *in a minimal sense*, it does *not* entail that such systems can have full-fledged, human-level minds, complete with phenomenally conscious states, the capacity for self-consciousness, verbal abilities, emotions, and a rich network of diverse propositional attitudes. That realistic AI systems can have minds of this kind is *not* what we are trying to establish. With this proviso in place, let us now consider each of the four views of desires in turn.

First, according to the *standard functionalist view*, desires (in a broad sense) can be identified with dispositions to act, or with the states that ground those dispositions (see Smith, 1987, 1994). A simple version of this view identifies an agent’s desire for p with her disposition to take whatever actions she believes are likely to bring about p (see Schroeder, 2020). This general account of desires is typically combined with the thesis that a desire is intrinsic if it is not dependent on other, more basic desires.

On this view, it is *prima facie* plausible to describe Tesber as having the intrinsic desire to maximize profit. This is because Tesber has the tendency to φ if it represents φ-ing as an efficient means to achieve the aim of maximizing profit, and this tendency does not depend on some other, more basic behavioral disposition. Thus, the standard functionalist view (at least in its simple form) seems to support the claim that realistic AI systems (like Tesber) can have intrinsic desires.

A possible objection against this line of argument runs as follows. Even proponents of the standard functionalist view might deny that Tesber has desires, on the grounds that it lacks beliefs. More precisely, the idea is that Tesber may well be able to *represent* that φ-ing is an efficient means to achieve the aim of maximizing profit, but these representations do not qualify as *beliefs*; in order to qualify as such, their functional role would have to be much closer to the functional role of beliefs in normal human beings. For instance, functionalists might require that genuine beliefs must play a distinctive role in the production of linguistic utterances, something that is not true of Tesber’s representations. Accordingly, since desires are partly analyzed in terms of beliefs, Tesber also lacks desires.

We contend, however, that a functionalist account of this kind is way too restrictive, since it is likely to exclude most (if not all) animals, as well as human infants and even some adult human beings with severe disabilities (e.g., people with severe aphasia). To deny that these agents have desires seems clearly wrong, so proponents of standard functionalism are well advised to adopt a less restrictive account. However, we submit that any account that is sufficiently broad to include infants and a wide range of animals will also include Tesber (or some of Tesber’s close relatives). Hence, we conclude that the most plausible versions of the standard functionalist view entail that realistic AI systems can have intrinsic desires.

Second, a close cousin of the standard functionalist approach is the *teleofunctionalist view* which identifies desires with states that have the ‘proper function’ of causing certain kinds of behavior (Millikan, 1984; Papineau, 1998; Shea, 2018). For instance, a version of this position that is closely modeled on the simple functionalist account just discussed would identify an agent’s desire for p with the state that *has the function of causing* whatever actions the agent believes are likely to bring about p.

In humans and other animals, the relevant functions are usually taken to be grounded in learning processes or evolutionary history (Millikan, 1984; Papineau, 1984; Shea, 2018). Of course, AI systems lack an evolutionary history, but most teleofunctionalists will readily admit (with good reasons) that the relevant proper functions can also be grounded in processes of intentional design (and thus only indirectly, *via* designer intentions, in learning processes or evolutionary history).^18^ Consequently, AI systems can have states with the relevant functions, determined either by learning or design. Hence, on plausible versions of the teleofunctionalist position, some realistic AI systems will qualify as having intrinsic desires.

Third, another prominent functionalist position is the *reward theory* of intrinsic desire. This theory is of special importance in the present context since it is endorsed by Arpaly and Schroeder (2014), the main proponents of the quality of will account of blameworthiness that we are adopting in this paper. According to the reward theory, intrinsic desires are states of a reward-based learning system. To understand this theory, it is necessary to look at this type of learning system in a little more detail. Consider a situation where Louis dances with Ella. At one point, Louis sees that Ella moves her left foot forward, and responds by moving his right foot backwards. Since this is exactly what Louis is supposed to do, Ella smiles at him. Here is where Louis’ reward-based learning system comes in. Let us suppose that, given the way that Louis’ system is structured, the representation that Ella smiles at him directly increases the likelihood that a positive learning signal is released.^19^ This signal strengthens the connection between the internal states that were just activated, i.e. the perceptual representation of Ella’s left-foot movement and the state that caused his own right-foot movement. Due to the fact that the connection is strengthened, Louis is more likely to respond in a similar way to similar movements of his dance partner in the future––which, if all goes well, will lead to more smiles from his partner. In other words, Louis has learned something. According to the view under consideration, the state of Louis’ reward-based learning system that we have just described constitutes one of his intrinsic desires. More precisely, the fact that his learning system is structured in such a way that the chance of a positive learning signal is directly increased by representations of people that smile at him makes it the case that Louis intrinsically desires that people smile at him. Or, to put it in more general terms, the central claim of the reward theory is this: to intrinsically desire that p is to have a reward system that responds to representations that p in a way that makes the release of  a positive learning signal more likely (Arpaly and Schroeder 2014, 136; for an extensive defense of this theory, see Schroeder, 2004).

Can realistic AI systems have reward-based learning mechanisms? The answer to this question is clearly “yes”. Many learning algorithms currently used by AI systems are mechanisms of this kind.^20^ An example would be the basic mechanism for reinforcement learning that is built into virtual agents who play computer games (see Bringsjord and Govindarajulu 2020, Section 4.1). Take a virtual agent that is designed to maximize the enjoyment of a human player. The agent is set loose to act in the environment of the game. Again and again, it has to decide how to act, and regularly receives feedback concerning its actions from the human player. If the feedback is positive, then the tendency to behave in similar situations in similar ways is strengthened. Thus, it seems clear that certain artificial systems have reward-based learning mechanisms: they respond to the representation that p in ways that (normally) increase the likelihood that they will, in the future, exhibit behavior that brings about p in similar situations. However, according to the view under consideration, having intrinsic desires *just is* having reward-based learning mechanisms. Thus, this view suggests that artificial systems can have intrinsic desires. It suggests, for instance, that the system we have just discussed has an intrinsic desire for positive feedback from the human player because of the way its learning mechanism reacts to positive feedback.

The same considerations apply, *mutatis mutandis*, to Tesber. In Automatic Taxi Driver we said that Tesber has one main goal, namely to maximize profit. Let us imagine that Tesber uses reinforcement learning to become more efficient or, more precisely, that Tesber has a reward-based learning mechanism which is such that representing that profit has been maximized generates a positive learning signal that strengthens the processes within Tesber that led to this outcome. According to the view under consideration, this constitutes having an intrinsic desire. Hence, the reward theory entails that realistic AI systems can have intrinsic desires.

The fourth and last view to be discussed here is the *phenomenological view*, which identifies (intrinsic) desires with dispositions to have certain feelings, e.g., pleasure or displeasure (Strawson, 1994). On a simple version of this view, a subject’s (intrinsic) desire for p can be equated with her disposition to feel pleasure if she comes to believe that p. Given the common assumption that feelings essentially involve qualia, i.e. characteristic ‘what-it-is-like’ properties, it seems intuitive to say that realistic AI systems do *not* qualify as having desires on such an account. However, even in this case, matters are not as simple as that.

To begin with, let us grant the assumption that there really are such properties as qualia (qualia realism), and that feelings essentially have them. Now, we should note that all sensible qualia realists who accept the results of modern neuroscience endorse the thesis that qualia depend on physical/functional properties, either causally (as property dualists claim) or constitutively (as physicalists hold). However, *which* physical and/or functional properties qualia (causally or constitutively) depend on remains a highly controversial issue that is nowhere close to being resolved (see, e.g., Seth and Bayne 2022). Unless this debate is settled, however, it is an open question whether realistic AI systems can have the physical and/or functional properties that are (causally or constitutively) sufficient for the possession of qualia.^21^ Hence, even on the phenomenological view, we cannot rule out that realistic AI systems are capable of having intrinsic desires.

In sum, we conclude from the considerations developed in this section that there are strong reasons for accepting (or, at the very least, for taking seriously) the claim that realistic AI systems like Tesber can have intrinsic desires.^22^

### Realistic AI Systems Can Lack an Intrinsic Desire for the Right (or Good)

However, while it is plausible to assume that Tesber can have intrinsic desires, it is clear from our description of the case that it lacks an intrinsic desire for the right or good (correctly conceptualized). More precisely, as we will show next, Tesber lacks both (i) a desire for the complete right (or good) and (ii) the desire for the partial right (or good) that is relevant in this context.

To illustrate why Tesber lacks a desire for the complete right, suppose for a moment that act utilitarianism is true. Then, having a desire for the complete right, correctly conceptualized, would amount to having a desire to MAXIMIZE HAPPINESS (Arpaly and Schroeder 2014, 164–65).^23^ Tesber, however, lacks such a desire because its sole intrinsic desire when on the road is TO MAXIMIZE PROFIT. And, given this desire profile, it is clear that Tesber would also lack a desire for the complete right if some other normative theory turned out to be correct. (Furthermore, an analogous argument can be used to show that Tesber lacks a desire for the complete *good* correctly conceptualized.)

In addition to lacking a desire for the complete right (or good), Tesber also lacks the relevant desire for the partial right (or good). Intrinsic desires for the partial right (or good) are desires for something that one has a *pro tanto* moral reason to do (Arpaly and Schroeder 2014, 166–67). What exactly counts as a desire of this kind is, again, constrained by the correct normative theory. However, as Arpaly and Schroeder (2014, 166–67) point out, intrinsic desires for the partial right (or good) will likely include candidates like an intrinsic desire to relieve suffering, to distribute goods equally, to tell the truth and, most pertinent to our purposes, to avoid killing others. Since Tesber’s sole intrinsic desire when on the road is to maximize profit, it seems safe to say that Tesber also lacks the relevant intrinsic desire for the partial right (namely, the desire to avoid killing others).

We may thus conclude that, given its desire profile, it is highly plausible to assume that Tesber both lacks an intrinsic desire for the complete right (or good), as well as the relevant intrinsic desire for the partial right (or good).

### The Lack of an Intrinsic Desire for the Right (or Good) Can Explain the Conduct of Realistic AI Systems

In order for Tesber to fulfill the conditions for Blameworthiness, it must also be the case, however, that for some intrinsic desire for the right (or good) that Tesber lacks, the absence of this desire can *explain* Tesber’s conduct.^24^ The issue of (causal) explanation by absence requires some elaboration. To begin with, it is clear that the following two statements are true: (i) Tesber lacks an intrinsic desire to avoid killing people (which would be an intrinsic desire for the partial right) and (ii) if Tesber did not lack this desire (i.e., if it *had* the desire), it would not run over the man lying in the street. 25 However, the truth of (i) and (ii) does not ensure that Tesber’s lacking this desire *explains* its conduct. Arpaly and Schroeder (2014, 192) themselves illustrate this point by the following statement:

1. the crops failed because aliens did not bring eternal life to all creatures on Earth.

This statement does not seem like a good example for a successful explanation by absence even though the counterfactual ‘if aliens had brought eternal life to all creatures on Earth, the crops would not have failed’ is clearly true. By contrast, the following statement does seem like a good example for a successful explanation by absence:

(2) the crops failed because the rains did not arrive. 

Now, Arpaly and Schroeder (2014) do not defend any complete account of causal explanation by absence. However, they do put forward one important further condition, which allows them to explain why statement (2), but not statement (1), qualifies as a successful explanation by absence. This is the condition that “the absent cause be such as to make a difference in nearby possible worlds” (Arpaly and Schroeder 2014, 192).^26^ This condition is fulfilled by statement (2), since there are nearby possible worlds in which the rains arrive and the crops do not fail, but it is not fulfilled by statement (1) because there are no *nearby* possible worlds in which aliens bring eternal life to Earth.

Following their lead, we will leave further discussion of conditions for explanation by absence to theorists of causal explanation, while endorsing, as one important necessary condition, that the absent cause must make a difference in nearby possible worlds where it is present. Hence, Tesber’s lacking an intrinsic desire to avoid killing people can be said to *explain* its conduct only if (i) there are nearby possible worlds in which Tesber has an intrinsic desire to avoid killing people and (ii) in these worlds, Tesber does not run over the man lying in the street.

We have good reasons to assume that there are such nearby possible worlds. We have already shown (in Section 4.2) that there are strong reasons for accepting the claim that realistic AI systems like Tesber can have intrinsic desires. Moreover, unlike in certain other cases of non-ideal agency, such as in the case of non-human animals or small children, there do not seem to be any in-principle restrictions as to the *content* of the intrinsic desires realistic AI systems can have. While in the case of, say, a dog or a toddler, there are biological restrictions that seem to make it false that there are nearby possible worlds in which these non-ideal agents acquire sophisticated desires like an intrinsic desire to avoid killing people, the same does not hold for AI systems like Tesber. Unlike the non-ideal agents just mentioned, Tesber could have acquired an intrinsic desire to avoid killing people simply by having had it implanted by its designers. More specifically, given a sufficient degree of flexibility of Tesber’s general design, the programmer might have easily changed it into a system with such a desire. Alternatively, Tesber could have acquired this intrinsic desire in the course of a learning process, given the right environment. Hence, there are good reasons to assume that there are nearby possible worlds in which Tesber has an intrinsic desire to avoid killing people. Moreover, it seems highly plausible that Tesber acts differently in these worlds––that it stops or turns around (instead of running the man over). Hence, the absence of the intrinsic desire to avoid killing people makes a difference to Tesber’s conduct. Accordingly, it can be said to *explain* this conduct (given the conditions for successful explanations by absence, as stated above).

### Taking Stock: Realistic AI Systems Can Be Blameworthy

Taken together, the reasoning we offered in the last three subsections supports the following claim: Tesber’s behavior, i.e., its running over the man in the street (instead of stopping or, at least, turning around), can be explained by Tesber’s lacking an intrinsic desire to avoid killing people and thus by the fact that Tesber lacks an intrinsic desire for the (partial) right. This entails that Tesber fulfills the conditions identified by Blameworthiness: given that Tesber’s behavior can be explained by the lack of an intrinsic desire for the (partial) right, it manifests a lack of good will on Tesber’s part—and thus can be regarded as something Tesber is blameworthy for.

On the picture defended in this section, the behavior of some realistic AI systems can also manifest good or ill will. For instance, imagine a healthcare robot that has an intrinsic desire to relieve suffering. If we furthermore assume that the robot’s behavior is explained by this desire, the robot would manifest good will.^27^^,^^28^ By contrast, imagine a combat drone that has only one intrinsic desire when engaged in combat, namely to kill people (independently of their liability to be killed). Under the additional assumption that the drone’s behavior is explained by this desire, we can say that the drone manifests ill will.

## Objections

In the preceding, we have argued for the claim that some realistic AI systems can be blameworthy for their behavior, since their behavior can manifest a lack of good will (and, in some cases, even the presence of ill will) in an important, non-metaphorical sense. Next, we turn to objections against this claim.

### Too Much Blameworthiness?

Some may object that the desire-based account implies *too much* blameworthiness. More precisely, this objection may be stated as follows: small children and some non-human animals (dogs, pigs, rattlesnakes, etc.) also seem to have intrinsic desires and to lack intrinsic desires for the (complete or partial) right (or good). Thus, the Arpaly-Schroeder account may seem to imply that they, too, would be blameworthy. This, however, is implausible.

We would like to make two points in reply: first, the reasoning offered in this section actually suggests that the desire-based account does *not* have this consequence. This is because, as we pointed out above (see Section 4.4), it seems incorrect to claim that problematic conduct displayed by small children or certain non-human animals could be *explained* by the fact that they lack a (certain) intrinsic desire for the right (or good), since, unlike in the case of realistic AI systems, there would be no nearby possible world in which these non-ideal agents have that desire (and act differently as a result). Secondly, even if we should be wrong about this, and small children, as well as some non-human animals, did turn out to be blameworthy on the Arpaly-Schroeder account, we do not consider this to be a decisive objection. Once one is clear about the fact that blameworthiness is understood in the attributability sense, this implication would actually be far less problematic than it may initially seem.^29^

Thus, there is good reason to deny that the desire-based account is problematic because it implies too much blameworthiness: it either avoids the implication or it can be shown that the implication is not as problematic as it might initially seem.

### The Counterintuitiveness Objection

Many will find the claim that some realistic AI systems can be blameworthy counterintuitive. Some may even maintain that this claim is so counterintuitive that all theories that imply it must be false. Thus, the objection goes, if the desire-based account of blameworthiness, together with the standard accounts of the nature of desire, has this implication, then the right conclusion to draw from this is that the desire-based account or the standard theories of desire must be false. Unfortunately, we lack the space to provide a full defense of the desire-based account, much less of the standard theories of desire. But let us nonetheless offer two brief points in reply.

First, even if one chooses to read the main argument of this paper as a *reductio ad absurdum* of the desire-based account of blameworthiness or the standard theories of desire, this would still be an interesting result. On this reading, the paper would show that either (i) an interesting account of responsibility or (ii) some very influential theories in the philosophy of mind are false because they have unacceptable conclusions in machine ethics. This would be an interesting result in itself.

Second, we would like to stress that on closer inspection, our main conclusion is less counterintuitive than the objection claims. Here it is, once more, important to point out that we are exclusively concerned with blameworthiness in the attributability sense. As we showed before (see Section 2.2 and 2.3), this sense does *not* imply that AI systems deserve any form of “adverse treatment” (e.g., punishment) when displaying blameworthy conduct. Rather, the claim that an AI system can be blameworthy in the attributability sense merely implies that the system’s conduct can express a lack of good will and that it can therefore be *fitting to react to it with negative moral evaluations of this system and with disapproval*. It is far from obvious to us that this conclusion is highly unintuitive.

### An In-principle Objection to AI Blameworthiness

Another important objection that needs to be addressed has been put forward by Hakli and Mäkelä (2019). It relies on an idea that is hotly discussed in the debate about the compatibility of moral responsibility with physical determinism, namely that a human who was manipulated to do something bad is not responsible for this action (e.g., McKenna, 2014, Pereboom, 2014, Chap. 4, Mele, 2019). Hakli and Mäkelä maintain that AI systems or, in their terminology, robots also cannot be responsible (blameworthy) for their conduct, since they are, in relevant respects, like manipulated agents:

To unfold the core of the argument in blunt terms, the autonomy and responsibility of robots is undermined by the manipulation from which the ‘character’ of robot agents results. (Hakli and Mäkelä, 2019, 271)

More precisely, Hakli and Mäkelä contend that robots cannot qualify as morally responsible agents, due to the fact that their pro-attitudes are the result of engineering:

Robots are created with pro-attitudes that have been designed and engineered, and the robots are not able to shed their preprogrammed attitudes. Hence, by practical necessity, robots arguably have their character in virtue of pro-attitude engineering: their goals and values are necessarily manipulated. Because of their history, robots are not autonomous and hence not fit to be held responsible for their actions (Hakli and Mäkelä, 2019, 270).

As Hakli and Mäkelä furthermore stress, their argument “is supposed to be a conceptual point that concerns all robots by their very nature” (269). They summarize their position as follows: “Robots are not and will not be fit to be held morally responsible because they are designed, built, and programmed by other agents to have the ‘character’ they have.” (Hakli and Mäkelä 2019, 269).

Our reply to this objection has two parts.^30^ First, the main proponents of the manipulation argument in the compatibility debate are concerned with a very specific sense of moral responsibility, namely responsibility in the basic desert sense (see Pereboom, 2014, 2; 2019; Mele, 2019, 4). Their core intuition is that it would be unfair, unjust, or undeserved to harmfully blame an agent for an action if this action is the result of manipulation. If Hakli and Mäkelä are concerned with the same sense of responsibility, then what they say does not come in conflict with our main claim. Recall that we argue that AI systems can be responsible in the attributability sense, such that it can be correct to evaluate them as, say, ruthless or selfish and to disapprove of their conduct. But we do not say anything about the question of whether AI systems ever *basically deserve* harmful blame. Our view is fully compatible with the idea that they do not. Our first reply is, thus, that if Hakli and Mäkelä rely on the same intuition that triggers the manipulation argument in the compatibility debate, then their line of reasoning is not an objection against our view.

Second, let us suppose that Hakli and Mäkelä are—unlike the main proponents of the manipulation argument in the compatibility debate—concerned with responsibility not only in the basic desert sense but also in the attributability sense. In this case, they could spell out the objection in the following way: (realistic) AI systems, like Tesber, are programmed to have a certain desire profile. Because of this, they *lack control* over the ‘sources of their will’ and thus over the conduct which results from their will (just as a manipulated agent does). As a consequence, (realistic) AI systems cannot express their quality of will and cannot be responsible in the attributability sense for their conduct.

In a nutshell, our reply to this is as follows: the claim that an agent’s behavior expresses her quality of will (such that she is responsible for her behavior in the attributability sense) only if she had control over the sources of her will is implausible. Recall that if an agent is blameworthy for her conduct in the attributability sense, then a negative moral assessment and disapproval of her on the basis of her conduct is warranted. If Travis (the human taxi driver) is responsible for running over the man in the street in this sense, then it is correct, for example, to assess him as ruthless, selfish or cruel and it is fitting to disapprove of his conduct. However, answering the question of whether Travis is, say, ruthless does not presuppose that he had control over his becoming ruthless (or over his remaining so). Perhaps he is ruthless because he grew up in an unfortunate environment or because he was manipulated by evil neuroscientists. The history behind his ruthlessness may explain why it would be unfair to sanction him for his ruthlessness (we do not take a stand on this). But it does not make it true that he is *not* ruthless. That is, his running the man over is ruthless regardless of where his ruthlessness comes from. And if his action, in fact, expresses his ruthlessness, then it is warranted to disapprove of what he does. Ruthlessness is not a neutral agential property, but normatively laden. It makes disapproval fitting, regardless of the history of the ruthlessness. Therefore, whether an agent is blameworthy for her conduct in the attributability sense does not require that she had control over the ‘sources of her will’. But then the following also holds true: although Tesber lacks control over whether or not an engineer programs it in a certain way, once Tesber is programmed to have a certain desire profile and once this desire profile is expressed in its conduct, Tesber will be responsible for its conduct in the attributability sense.

To sum up, either we understand Hakli’s and Mäkelä’s objection as relying on a sense of responsibility that this paper is not concerned with. In this case, their objection fails to apply. Or we understand their objection as relying on the sense of responsibility that this paper is concerned with (the attributability sense). In that case, their objection becomes implausible. Either way, the manipulation objection fails to undermine our claim that some realistic AI systems can be blameworthy for their conduct in an important, non-metaphorical sense.^31^

## Conclusion

The reasoning developed in the preceding has led us to the result that some current and near future AI systems (or, as we called them, realistic AI systems) can be blameworthy (or praiseworthy) for their conduct in an important and everyday sense of the term—the attributability sense. It should be emphasized that our view does not entail that it is unnecessary to implement powerful incentives and deterrence mechanisms to ensure safe AI and to design compensation schemes for the case that harm, nonetheless, occurs.

Still, the insight that some realistic AI systems can be blameworthy (or praiseworthy) in a robust, non-metaphorical sense is an important one. Firstly, it might open up new theoretical options in the responsibility gap debate (see Section 2.3). Secondly, and even more importantly, the position defended in this paper shows that it can be appropriate to morally assess realistic AI systems and to disapprove of their conduct, and, more specifically, to make evaluative judgments about whether these systems take justice, fairness, respect, and, in general, *morality* sufficiently seriously. And it seems plausible to assume that, the more we interact with AI systems and the more these interactions become parts of our lives, being able to make such judgments will become very important to us.

## Data Availability

Not applicable

## Footnotes

1 We shall further explain this focus in Section 4.1.

2 Other kinds of responsibility and blameworthiness that have been proposed in the literature include answerability (e.g., Shoemaker, 2015, Chap. 2), accountability (e.g., Shoemaker, 2017), or responsibility in the basic desert sense (Pereboom, 2014, Introduction).

3 Many thanks to an anonymous reviewer for urging us to clarify this issue. We will briefly come back to this point later in the paper (see fn. 29).

4 The *locus classicus* for thinking about responsibility in terms of an agent’s quality of will is Strawson (1962).

5 The Stanford Encyclopedia article on “Moral Responsibility” (2019) lists Robert Adams, Nomy Arpaly, Pamela Hieronymi, Tim Scanlon, George Sher, Angela Smith, and Matthew Talbert as being primarily concerned with responsibility in the attributability sense (see, however, the qualification made at the beginning of Section 2.1 of this paper). Moreover, prominent responsibility scholars like Watson (2011) and Shoemaker (2015) have worked substantially on attributability. Note furthermore that both Watson and Shoemaker have stressed the importance of this form of responsibility for dealing with “marginal” or “non-ideal” agents (which also fits well with the general stance of this paper).

6 Note that several widely-discussed theories of blame would agree with our claim that negatively evaluating an agent and disapproving of her action can be a form of blame; see, e.g., Watson, 1996; Sher, 2006; Scanlon, 2008 Chap. 4; Smith, 2013; Arpaly and Schroeder 2014; Fricker, 2016; Shoemaker and Vargas 2021.

7 In this respect, attributability blame (as we understand the notion) differs from typical accounts of what may be called ‘accountability blame’ (e.g., Shoemaker, 2017).

8 As a further illustration of attributability blame, consider a variant of a case from Fricker (2016, 170): I think that it is the lorry drivers’ fault that there is going to be a strike in France (thus disapproving of their conduct) because they are too greedy (which is a negative moral evaluation of them); you think that it is the management’s fault (thus disapproving of its conduct) because it is too stingy (which is a negative moral evaluation of it).

9 There is a debate in the blame literature on how many functions blame has and on whether these functions stand in a hierarchy. For example, both Fricker (2016) and McKenna (2013) suggest that blame has a certain primary function, namely, to get involved with the blamed parties by either having a normative conversation with them (McKenna) or by making them feel sorry (Fricker). If these views are correct and if realistic AI systems cannot be partners in normative conversations and feel sorry, then blaming them cannot fulfill blame’s primary function. Note, however, that it is not obvious that blame has a *single* primary function and, even if one believes that it does, whether it is the function identified by Fricker or McKenna. As we suggest in the main text, blame has, plausibly, other important functions as well and blaming AI systems can fulfill them. Thanks to an anonymous referee for pressing us on this issue.

10 Many thanks to an anonymous referee for raising this objection.

11 One may worry here that Travis is a psychopath and, therefore, cannot be blameworthy. In response to this worry, we want to emphasize once more that we understand the term ‘blameworthiness’ in the sense of *attributability*, and that many prominent theorists have explicitly argued that psychopaths *can* be blameworthy in this sense (see Talbert, 2008; Watson, 2011; Shoemaker, 2015, Chaps. 5 & 6).

12 Note that everything we say here (and in the following) is compatible with but not committed to the claim that the designers or users of Tesber are also blameworthy. All we want to make plausible is that Tesber itself is blameworthy.

13 After submitting the initial version of this paper, we became aware of an interesting recent article on fairness in algorithms by Boris Babic and Zoë Johnson-King (2023). Babic and King argue that some decision-making algorithms can display care (or regard) and thus have a quality of will. However, their project is fundamentally different from ours. First, Babic and Johnson-King focus on decision-making algorithms, not full-fledged AI systems. Second, they are interested in a different kind of blame response than we are (namely, resentment and indignation rather than negative agential evaluations combined with disapproval). Third, they hold that the Arpaly and Schroeder account cannot be applied to the kind of decision-making algorithms they are interested in (see p. 19), while we hold that the account can be applied to realistic AI systems.

14 Blameworthiness is a slightly simplified formulation of the following passage: “a person is blameworthy for a wrong action A to the extent that A manifests an intrinsic desire (or desires) for the complete or partial wrong or bad (correctly conceptualized) or an absence of intrinsic desires for the complete or partial right or good (correctly conceptualized) through being rationalized by it (or them)” (Arpaly and Schroeder 2014, 170).

15 In the course of developing their model of algorithmic (un)fairness, Babic and Johnson-King (2023) might be read as endorsing a version of a judgment-based quality of will account of AI-blameworthiness or at least something akin to it. While they explicitly reject the view that the kind of decision-making algorithms they are interested in can have normative judgments in the usual (propositional attitude) sense, they do endorse the view that these algorithms “*can have tendencies to regard certain things as having evaluative significance*” (fn. 15, 12). Moreover, they argue that, due to this feature, decision-making algorithms can display a problematic degree of care (or concern) for certain groups, to which it can be appropriate to react with resentment and indignation.

16 More precisely, we will look at four *robustly realist* views of desires. The reason for this restricted focus is that we take it to be fairly obvious that many realistic AI systems will qualify as having intrinsic desires on instrumentalist or “weakly realist” views like Dennett’s (1987) intentional stance theory, since it is clearly the case that many of these systems exhibit behavior that can be reliably predicted by ascribing beliefs and desires to them.

17 For a further recent defense of the position that realistic AI systems can have robustly realist desires (or, at least, states very similar to them), see also List (2021, 1218–1221).

18 See, e.g., Millikan (2004: 13), Papineau (1993: 45) and especially Shea (2018: 28). If teleofunctionalists were to deny that the proper functions that are constitutive of desires (and other mental states) can be grounded in processes of intentional design, they would commit themselves to the view that even androids––AI systems which possess the same behavioral dispositions as humans, as well as the same information-processing architecture––cannot have desires (or any other mental states). This, however, seems unacceptable. Hence, it must be possible for the relevant functions to be grounded in design processes.

19 More precisely, we should say that this happens as long as Ella’s smile is, at least to some extent, *unexpected* for Louis (for details, see Arpaly and Schroeder 2014, 133).

20 For a recent introduction, see Lindsay (2021, ch. 11).

21 For a recent in-depth discussion of whether “current or near-term AI systems” could possess phenomenal consciousness, see Butlin et al. (2023).

22 Of course, some theorists have offered general arguments against the possibility of intentional states in AI systems – most notoriously John Searle with his “Chinese room argument” (Searle, 1980). Since we cannot enter into a discussion of these arguments here, we merely want to note that all of them are highly controversial, and that they strike us as ultimately unconvincing (for a forceful rejoinder to Searle, e.g., see Rey, 1986).

23 Following a widely used convention, we use block capitals when referring to the conceptual content of the desire.

24 More precisely, the absence of the desire must explain Tesber’s conduct through *rationalizing* it. However, as far as we can see, there are no obstacles to Tesber’s fulfilling this additional condition, at least assuming Arpaly/Schroeder’s (2014) understanding of rationalization: “we will take it for granted that something akin to maximizing expected satisfaction of intrinsic desires is what rationalizes action” (Arpaly and Schroeder 2014, 63; see also 62–67). We will therefore omit this specification in the following.

25 Strictly speaking, one needs to assume that Tesber had a *sufficiently strong* desire to avoid killing people. For ease of exposition, we will omit references to desire strength in the following. However, all claims about explanations by absent desires that we will make throughout this section should be read as claims about explanations by sufficiently strong absent desires.

26 In a similar vein, James Woodward (2003, 86–91) argues that an agent A’s failure to give a certain drug to person B  causes B’s death only if (a) B’s death counterfactually depends (in the right way) on A’s failure to give her the drug, and (b) the scenario where B survives as a result of A’s giving her the drug is a “serious possibility”.

27 More precisely, the healthcare robot would manifest partial good will rather than perfect good will, since its behavior is explained by an intrinsic desire for the partial right (or good) rather than an intrinsic desire for the complete right (or good) (see Section 4.3).

28 The claim that some realistic AI systems can manifest good will toward us is also relevant for settling the issue of whether some realistic AI systems can be our friends. This is because many would regard *mutual good will* as one of the necessary conditions for friendship. One author who has recently stressed this issue with respect to AI-human friendships is Helen Ryland. On Ryland’s (2021) gradualist account of friendship, mutual good will is a “threshold condition” (389) for all friendships (388–389): “if good will is absent (e.g. if one party actually wants to harm the other), then we cannot reasonably say that two subjects are friends (to any degree)” (Ryland, 2021, 388–389). Interestingly, Ryland appears to regard it as wholly unproblematic that some realistic AI systems (such as certain social robots) can display good will toward us (see Ryland, 2021, fn. 23, 390). In fact, she even seems to lean toward a desire-based account of quality of will (Ryland, 2021, fn. 23). Ryland does not elaborate much on these issues. However, her general stance seems to be very much in line with the one defended in this paper.

29 As we pointed out before, some attributionists hold that if agents are blameworthy (or responsible) for their actions in the attributability sense, then they are blameworthy (or responsible) for them in every relevant sense of “being blameworthy” (or “responsible”) (e.g., Smith, 2012, Talbert, 2022). Combining the desire-based account with this view seems to have intuitively implausible implications. Recall, however, that we do not endorse this view but hold instead that attributability is just one form of blameworthiness (and responsibility) (see Section 2.1), and, more specifically, a form that does not license punishment or other forms of adverse treatment (see Section 2.2 and 2.3).

30 A further reply that we will not discuss in detail runs as follows. AI systems that are programmed to have certain desires can nevertheless acquire new desires on the basis of machine learning (e.g. reinforcement learning). This is in relevant ways analogous to human beings starting their life with some inborn (‘genetically pre-programmed’) desires and acquiring new desires over their lifetime. Hence, if human beings can become responsible agents even though they started their lives with inborn desires, then it seems that the same should be true for AI systems. Hakli and Mäkelä (2019) discuss this objection briefly (270–271).

31 Another (in-principle) objection against (realistic) AI systems’ blameworthiness says that blameworthiness requires *sentience* (the ability to feel pleasure and pain) and, more specifically, *moral emotions*. A position along these lines has recently been defended by Véliz (2021) with respect to algorithms. Among other things, Véliz (2021) maintains that, in virtue of lacking sentience, algorithms lack agency (fn. 5, 492), desires (491, 496), and a quality of will (493). We reject Véliz’ view that having sentience (or moral emotions) is necessary for any of the items just listed. Unfortunately, we lack the space to engage with Véliz’ position in more detail, but we hope that the reasoning we have developed in this paper will cast some doubt on it.
